# Prevalence, correlates for early neurological disorders and association with functioning among children and adolescents with HIV/AIDS in Uganda

**DOI:** 10.1186/s12888-019-2023-9

**Published:** 2019-01-21

**Authors:** Richard Stephen Mpango, Godfrey Zari Rukundo, Sylvia Kiwuwa Muyingo, Kenneth D. Gadow, Vikram Patel, Eugene Kinyanda

**Affiliations:** 1 Department of Psychiatry, Mental Health Project, MRC/UVRI and LSHTM Uganda Research Unit, Makerere College of Health Sciences, P. O. Box 49, Entebbe, Uganda; 2 Department of Psychiatry, Mbarara University of Science and Technology, P. O. Box 1410, Mabarara, Uganda; 3 Statistical Section, MRC/UVRI and LSHTM Uganda Research Unit, Entebbe, Uganda; 4 Department of Psychiatry, Health Sciences Centre, Stony Brook University, Stony Brook, New York, NY 11794-8790 USA; 5 Department of Global Health and Social Medicine, Harvard Medical School, Boston, MA USA; 6 Mental Health Project, MRC/UVRI and LSHTM Uganda Research Unit, P. O. Box 49, Entebbe, Uganda

**Keywords:** Neurological disorders, Children/adolescents, HIV, Prevalence, Correlates

## Abstract

**Background:**

The aim of this study was to determine the prevalence of neurological disorders and their associated correlates and relations with clinical and behavioural problems among children and adolescents with HIV/AIDS (CA-HIV).

**Methods:**

This study involved a sample of 1070 CA-HIV/caregiver dyads who were evaluated at their 6-month follow-up visit as part of their participation in the longitudinal study, *‘Mental health among HIV infected CHildren and Adolescents in KAmpala and Masaka, Uganda (the CHAKA study)’.* Participants completed an extensive battery of measures that included a standardized *DSM-5-* referenced rating scale, the parent version (5–18 years) of the Child and Adolescent Symptom Inventory-5 (CASI-5). Using logistic regression, we estimated the prevalence of neurological disorders and characterised their associations with negative clinical and behavioural factors.

**Results:**

The overall prevalence of at least one neurological disorders was 18.5% (*n* = 198; 95% CI, 16.2–20.8). Enuresis / encopresis was the most common (10%), followed by motor/vocal tics (5.3%); probable epilepsy was the least prevalent (4%). Correlates associated with neurological disorders were in two domains: socio-demographic factors (age, ethnicity and staying in rural areas) and HIV-related factors (baseline viral load suppression). Enuresis/encopresis was associated with psychiatric comorbidity. Neurological disorders were associated with earlier onset of sexual intercourse (adjusted OR 4.06, 95% CI 1.26–13.1, *P = 0.02*).

**Conclusions:**

Neurological disorders impact lives of many children and adolescents with HIV/AIDS. There is an urgent need to integrate the delivery of mental and neurological health services into routine clinical care for children and adolescents with HIV/AIDS in sub-Saharan Africa.

**Electronic supplementary material:**

The online version of this article (10.1186/s12888-019-2023-9) contains supplementary material, which is available to authorized users.

## Background

Children and adolescents infected with HIV/AIDS (CA-HIV) are at increased risk of developmental and neuropsychological disturbances owing to both the direct and indirect effects of the HIV virus [1, 2]. HIV has direct neurotoxic effects on brain structures involved in the regulation of emotion, behavior, and cognition, this effect is thought to result primarily from HIV’s ability to induce inflammatory factors that cause neuronal cell damage and eventual cell death [1]. The indirect effects of HIV include social stressors, poverty, illness and trauma on morbidity and mortality [1, 2]. Previous research suggests that HIV-1 mutations differ by subtype, which may lead to increased resistance to anti-retroviral therapy (ART) thus resulting in persistence of neurological and neurodevelopmental disorders among CA-HIV receiving ART [2].

In sub Saharan Africa, with the exception of epilepsy, few studies have attempted to determine the prevalence of neurologic disease among children and adolescents and therefore characterize their clinical correlates [1]. In one study conducted in East Africa, approximately two-thirds of new patients presenting to a child and adolescent psychiatry service had epilepsy [3], and seizures are also common among individuals with HIV/AIDS [4]. Although neurological disorders are reported to be associated with HIV/AIDS [1, 2, 5], no such studies have been conducted among CA-HIV in Uganda. The primary objectives of the present study were to (a) document the prevalence of neurological disorders among a large sample of CA-HIV attending rural and urban HIV clinics in Uganda, (b) characterize the relation of neurological disorders with a wide range of commonly studied clinical correlates, (c) establish the co-occurrence of neurological and psychiatric disorders, and (d) investigate the relation of neurological disorders with important indices of functioning.

## Methods

### Participants

The study sample comprises 1070 CA-HIV and their caregivers who were evaluated at their 6-month follow-up visit as part of their participation in the *Mental health among HIV infected CHildren and Adolescents in KAmpala and Masaka, Uganda (CHAKA) study.* Details about the study design, eligibility criteria and study procedures of CHAKA are described in a previous publication [6]. Of the initial 1339 CA-HIV/ caregiver dyads interviewed at baseline, 269 (20.0%) were not included in the 6 months analyses for various reasons including: 3 had incomplete records and the remaining 266 CA-HIV did not turn up for the 6 months follow-up interview despite various attempts to reach them. Some of these later returned for their 12-month interview reporting that they had temporarily transferred out of the study clinic.

### Measures

**Fig. 1:**
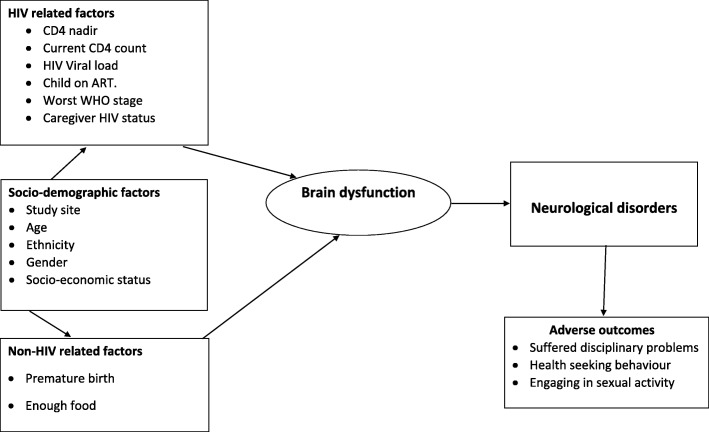
Conceptual framework for clinical correlates of neurological disorders

The assessment battery comprised locally adapted standardised psychosocial instruments described in earlier publications [6, 7] that have previously been used in the USA [8–10]. Study variables reported in this paper are described in Additional file 1 and are arranged according to the conceptual framework depicted in Fig. 1.

#### Epilepsy

Probable clinical epilepsy was based on a score of *yes* to the following item: *Has this child/adolescent ever suffered repeated seizures or convulsions without fever being present?*

#### Tic and elimination disorders

Motor tics and vocal tics and elimination disorders (enuresis and encopresis) were established using a clinically practical, DSM-5-referenced, behaviour rating scale, the Child and Adolescent Symptom Inventory-5 (CASI-5) [10], which was previously used to study emotional and behavioral disorders among CA-HIV in the United States [9, 11]. The items in the CASI-5 are based on symptom statements that appear in the Diagnostic and Statistical Manual of Mental Disorders, Fifth Edition (DSM-5). Informants indicate whether these symptoms occur, *never*, *sometimes*, *often*, or *very often*. CASI-5 items can be scored in several different ways, and in the present study we used the symptom count cut-off score for these items. Symptom count cut-off score indicates whether child/adolescent has the prerequisite number of symptoms necessary for a DSM-5 diagnosis, which for this study was a rating of *often* or *very often*.

### Psychiatric disorders

The CASI-5 also provides scoring algorithms for estimating psychiatric diagnoses. For purposes of this paper, we generated clinical cutoff scores for 12 psychiatric disorders, which we categorised as either internalising or externalising. The internalising disorder category included generalised anxiety disorder, social anxiety disorder, separation anxiety disorder, specific phobia disorder, panic disorder, post-traumatic stress disorder (PTSD), major depressive episode (MDD and dysthymia). Externalising disorders included attention-deficit/hyperactivity disorder (ADHD), oppositional defiant disorder (ODD), conduct disorder, and callous unemotional. To receive a clinical cutoff score, a CA-HIV had to meet both DSM-based symptom count criteria for a specific disorder as well as impairment cutoff criteria. The latter is a rating of *often* or *very often* to the question of whether symptoms interceded with social, academic, or chore-related functioning. The CASI-5 was administered by trained psychiatric nurse research assistants.

#### Sexual intercourse

Sexual intercourse was a response of *yes* to the question, *Have you ever had sex?* Possible responses were yes/no*,* this question was asked only to adolescents.

### Statistical analysis

Prevalence estimates and 95% confidence intervals for the three neurological outcomes of enuresis/encopresis, motor or vocal tics and probable epilepsy are reported overall and by age and gender category. Using a conceptual framework (Fig. 1) based on both HIV- and non-HIV-related factors hypothesized to be associated with neurological disorders, we investigated several widely studied variables. Associations between each of the neurological disorders evaluated at the 6-month follow-up period, baseline socio-demographic and HIV-related factors were undertaken using logistic regression models. Multivariable models were selected based on backward elimination, and models were fitted sequentially to include significant factors from each category at *p* < 0.1. The final model included the pre-defined design variable of study site, age and sex and all significant factors from the previous models.

The prevalence and 95% confidence intervals of comorbidities between neurological disorders as well as externalising and internalising disorders were estimated. We investigated associations of neurological disorders with the following outcomes using logistic regression: social outcomes as evidenced by disciplinary actions in school, any missed days at school during the last term, and onset of sexual intercourse (yes/no; adolescents only); and health-related outcomes such as any visit to the health unit in the last month; any hospital admissions in the last 6 months, and non-adherence to ART defined as any missed ART in the last 3 days (yes/no). For each outcome, associations were assessed using logistic regression models, adjusted for age, sex, study site and ethnicity. In accordance with previously published work from the CHAKA study, adjustment was made for CD4 cell count, visits to the health unit, and hospital admissions and for HIV ART status for the adherence outcome. Similarly, we used 5% *p*-values for these descriptive exploratory analyses because it is not clear how many comparisons should be corrected for and precautions taken in interpretation due to multiple comparisons. We used Bonferroni correction adjusted *p*-values by multiplying the observed *p* value from the significance tests by the number of tests k, for each of the analyses with psychiatric symptom outcomes [26, 27]. Then if any kP is less than 0.05 the differences in the two groups (with versus those without neurological disorders) are significant at the 0.05 level.

## Results

### Characteristics of study participants

Overall, the study included 677 (63.3%) children aged 5–11 years and 393 (36.7%) adolescents aged 12–17 years. The urban and rural study sites contributed equally. There were slightly more females (52%) than males (48%). With regard to HIV illness parameters, 88% had CD4 counts equal or greater than 350 cells/μL, and most (95%) of the participants were receiving ART (Additional File 2).

### Prevalence of neurological disorders

One hundred ninety eight participants had at least one neurological disorder (prevalence 18.5%). The prevalence of neurological disorders was 10% for enuresis/encopresis, 5.3% for motor/vocal tics, and 4% for probable epilepsy (Table 1). Of the 198 with any neurological disorder, 189 (17.7%) had at most one disorder [tics (*n* = 51, enuresis/encopresis (*n* = 102), and epilepsy (*n* = 36)], and 9 (0.8%) had at most two disorders [tics and enuresis/encopresis (*n* = 2), tics and epilepsy (*n* = 4), enuresis/encopresis and epilepsy (*n* = 3)]. The prevalence of any neurological disorder was 18% in males and 19% in females. The prevalence of probable epilepsy among children and adolescent was similar (4.0% vs. 4.1%), and enuresis/encopresis was higher among children than adolescents (14.0% vs. 3.1%), whereas motor/vocal tics were more frequent among adolescents than children (11% vs. 2%). Marginal gender differences in prevalence of probable epilepsy in females versus males (5% vs 3%) and in enuresis/encopresis in females versus males (9% vs 11%), whereas motor/vocal tics were more common in females versus males (7% vs 4%).

**Table 1:** Prevalence and 95% CI of neurological disorders based on symptom counts cut-off scores of the CASI-5

Subgroup	N	Prevalence enuresis/encopresis n (%) 95% CI	Prevalence motor/vocal tics	Prevalence epilepsy	Prevalence any neurological disorder
Children	677	95 (14.0%) (11.4, 16.7)	14 (2.1%) (1.0, 3.1)	27 (4.0%) (2.5, 5.5)	131 (19.4%) (16.4, 22.3)
Adolescents	393	12 (3.1%) (1.3, 4.8)	43 (10.9%) (7.8, 14.0)	16 (4.1%) (2.1, 6.0)	67 (17.1%) (13.3, 20.8)
Female	550	48 (8.7%) (6.4, 11.1)	37 (6.7%) (4.6, 8.8)	26 (4.7%) (3.0, 6.5)	106 (19.3%) (16.0, 22.6)
Male	520	59 (11.3%) (8.6, 14.1)	20 (3.9%) (2.2, 5.5)	17 (3.3%) (1.7, 4.8)	92 (17.7%) (14.4, 21.0)
Overall	1070	107 (10%) (8.1, 11.8)	57 (5.3%) (4.0, 6.7)	43 (4.0%) (2.8, 5.2)	198 (18.5%) (16.2, 20.8)

### Correlates of elimination disorders

Factors associated with enuresis/encopresis symptom count cut-off scores in the multivariable model were younger age (aOR 0.79, 95% CI 0.73–0.85, *P < 0.001*), ethnicity (being a non-Muganda with aOR of 0.56, CI 0.33–0.95, *P = 0.03*), living in rural areas (aOR 2.25, CI 1.42–3.54, *P = 0.4*), and less viral load suppression at baseline (aOR 1.75, CI 1.08–2.83, *P = 0.02*) (Additional file 3).

### Clinical correlates of motor and vocal tics)

CA-HIV socio-demographic variables associated with motor/vocal tics symptom count cut off scores in the multivariable model were increasing age (aOR 1.28, CI 1.17–1.40, *P < 0.001*) and ethnicity (being non-Muganda; aOR 0.33, CI 0.14–0.76, *P = 0.01)* adjusted for site and sex (Additional file 3).

### Clinical correlates of probable epilepsy

We found ethnicity marginally associated with probable epilepsy (being a non-Muganda with aOR of 1.87, CI 0.98–3.57, *P = 0.06*), adjusted for age, sex and site. None of the other factors was associated with probable epilepsy (*p > 0.1*) (Additional file 3).

### Association with psychiatric symptoms

CA-HIV with enuresis/encopresis had a higher risk for co-occurring externalising disorder (18.7%) and internalising disorder (36.4%) (*p < 0.001*, and *p* < 0.001, respectively) (Table 2). All the other associations between neurological disorders and psychiatric comorbidities were not significant.

**Table 2:** Comorbidities between neurological disorders with externalising and internalising psychiatric disorders

*N* = 1070	Enuresis/ Encopresis n(%) CI, *p*-value	Motor/vocal tics	Epilepsy	Any neurological disorder
n	107	57	43	198
Externalising disorder	20 (18.7%)	5 (8.8%)	4 (9.3%)	28 (14.1)
	(12.4, 27.3)	(3.6, 19.5)	(3.6, 19.5)	(9.9, 19.7)
	***P*** **< 0.001**	***P*** **= 0.9**	***P*** **= 0.9**	***P*** **= 0.01**
Internalising disorder	39 (36.4%)	14 (24.6%)	14 (32.6%)	63 (31.8%)
	(27.8, 46.0)	(15.0, 37.4)	(20.1, 25.2)	(25.7, 38.7)
	***P*** **< 0.001**	***P*** **= 0.8**	***P*** **= 0.1**	***P*** **= 0.001**

Externalising includes; any ADHD, ODD, CD, callous unemotional by symptom frequency, or clinically significant or interferes with function

Internalising; MDD, persistent dysthymia, GAD, panic disorder, specific phobia, PTSD, any social anxiety, separation anxiety,

### Association with social and academic functioning

Having *any* neurological disorder was associated with an earlier onset of sexual intercourse (adjusted OR 4.06, 95% CI 1.26–13.1, *P = 0.02*). The relation of neurological disorders with the other social or academic outcomes were not significant (Table 3).

**Table 3:** Association between neurological disorders and adverse outcomes

Outcomes	Enuresis/ Encopresis aOR, *p*-value^a^	Motor/ Vocal TICS aOR, *p*-value^a^	Epilepsy aOR, *p*-value^a^	Any neurological disorder aOR, *p*-value^a^
Any poor social functioning (Suffered disciplinary measures ie suspension, dismissal)^f^	0.73 (0.16, 3.27)	0.71 (0.23, 2.15)	1.10 (0.43, 2.77)	1.06 (0.31, 3.61)
	***P = 0.7***	***P = 0.5***	***P = 0.8***	***P = 0.9***
Any days missed at school in the last term^f^	0.89 (0.59, 1.35)	0.93 (0.67, 1.30)	1.35 (0.99, 1.85)	1.17 (0.78, 1.74)
	***P = 0.6***	***P = 0.7***	***P = 0.06***	***P = 0.4***
Any visit to the health unit in the last month^c,f^	O.44 (0.17, 1.13)	0.57 (0.30, 1.06)	0.98 (0.59, 1.63)	1.39 (0.76, 2.56)
	***P = 0.09***	***P = 0.08***	***P = 0.9***	***P = 0.3***
Any admission to hospital in last 6 months^c,6^	0.66 (0.15, 2.91)	0.94(0.34, 2.60)	0.85 (0.34, 2.13)	1.29 (0.44, 3.79)
	***P = 0.6***	***P = 0.9***	***P = 0.7***	***P = 0.6***
Early onset of sexual intercourse^b,e,f^	N/A	0.78 (0.26, 2.30)	1.87 (0.56, 6.21)	4.06 (1.26, 13.1)
		***P = 0.7***	***P = 0.3***	***P = 0.02***
Any non-adherence to ART in last 3 days^b,d,f^	N/A	0.78 (0.33, 1.85)	1.21 (0.46, 3.21)	1.60 (0.55, 4.66)
		***P = 0.6***	***P = 0.7***	***P = 0.4***

^a^Adjusted for age, sex, study site and ethnicity

^b^only asked in adolescents,

^c^Adjusted for CD4 cell count at baseline

^d^Non adherence to ART adjusted for CD4 count at baseline and whether CA-HIV on ART

N/A – outcome does not vary

^e^Bonferroni adjusted *p* value is 0.02*3 = 0.06

^f^Bonferroni adjusted p value is > 0.1

## Discussion

This study aimed to determine the prevalence of neurological disorders among CA-HIV in Uganda and their association with commonly studied mental health clinical correlates, associated psychiatric comorbidities, and functioning. The prevalence of at least one neurological disorder among CA-HIV was 18.5%, which is consistent with findings from previous studies that suggest a high prevalence of neurological disorders among CA-HIV [2, 4].

The prevalence of probable epilepsy in this study was 4%, which is within the range reported in prior studies both in the West and in South Africa. Kellinghaus and colleagues (2008) found that among HIV positive adult patients attending a neurological clinic in Germany, 6.1% had epilepsy [12]. Pascual-Sedano and colleagues (1999) reported a 3% prevalence for new-onset seizures during a one year follow-up period among an adult HIV cohort in Spain [13]. In one of the few studies in this area from sub-Saharan Africa, Samai and colleagues (2013) noted that among HIV positive youth in South Africa, 7.6% had epilepsy [14].

The prevalence of enuresis/encopresis in this study was 10%, and we were unable to locate any other studies in the extant literature about these problems among CA-HIV for purposes of comparison. A rate of enuresis of 5–9% [15–17] and that of encopresis of 1–3% [15–17] have been reported among general population samples of children. In this study, the prevalence for motor/vocal tics was 5.3%, a figure within the 4–12% range reported among children in the general population [18].

In this study, the only variable that was marginally associated with probable epilepsy was ethnicity. Belonging to an ethnic group that was not indigenous to the study area conferred an increased risk for epilepsy. Ethnicity could be associated with epilepsy for two reasons. Firstly, ethnicity may be a pointer to the genetic underpinnings of epilepsy [19]. Secondly, ethnicity may be a marker for socio-economic disadvantage with respondents who were not indigenous to the study area being more likely to experience medical complications while growing up that are associated with both social disadvantage and epilepsy such as birth trauma, other central nervous system infections (including complicated malaria), and traumatic brain injury [20].

In this study, enuresis/encopresis were significantly associated with younger age and marginally higher among males than in females. Both enuresis and encopresis have previously been reported to decrease with increasing age [16, 21, 22] and to be higher among males than among females [21, 22]. In this study, enuresis/encopresis was significantly associated with living in a rural area. Living in a rural area was probably an index of low socio-economic status. Indeed, in a Turkish study [16], enuresis was reported to be associated with low maternal education and with low monthly income, indices of poor socio-economic status. Ethnicity was significantly associated with enuresis/encopresis in this study. As previously discussed under probable epilepsy, the association between enuresis/encopresis and ethnicity may be underlined by the same reasons namely, as a pointer to an underlying genetic underpinning for enuresis/encopresis [23], secondly, as a marker for socio-economic disadvantage. The association between enuresis/encopresis and ‘less HIV viral load suppression’ observed in this study may be due to the direct neurotoxic effect of HIV or/ and to the increased psychological distress associated with a worse HIV clinical state. Lastly, enuresis/encopresis were the only neurological disorders significantly associated with both externalising and internalising psychiatric disorders. Previous research has reported that 20–40% of all children with enuresis have additional comorbid psychiatric disorders which include the externalising disorders of attention deficit hyperactivity disorder (ADHD) and oppositional defiant disorder (ODD) and the internalising disorder of depression [21]. In a large community study, children with encopresis were reported to have increased rates of the externalising disorders of ADHD and ODD and the internalising disorders of separation anxiety, specific phobia and generalised anxiety disorders [24].

In this study motor/vocal tics were more common in females than in males. Scahill and colleagues (2014) in a review involving eleven community studies reported great variation in the female to male ratio of motor/vocal tics ranging from 1 to 1, 1 to 4, through to 1 to 10 [25]. The factors significantly associated with motor/ vocal tics in this study included increasing age and ethnicity. Metzger and colleagues (2012) observed that as children progress into young adulthood, tics often go into remission [18]. The significant association between motor/vocal tics and ethnicity as discussed under probable epilepsy is probably underlined by two factors, a genetic underpinning [26] and social disadvantage.

Having any neurological disorder in this study was associated with an earlier onset of sexual intercourse. The reasons for this association were not immediately clear, however this relationship could be mediated through psychiatric comorbidity [6].

This study has several strengths that include a large sample size, comprehensive assessment battery, and cross-cultural orientation, however, there were also several limitations. Firstly, it is not possible to comment on the casual direction of obtained associations given the cross-sectional design. Secondly, neurological disorders were assessed using single-item questions which increased the risk of false positives. Thirdly, because we did not have a comparable sample of sero-negative youth from the same geographic areas, it is not possible to know whether relations between neurological and putative mental health factors and functional outcomes are influenced by HIV status. This does not, however, detract from the clinical implications of our findings for CA-HIV. Lastly, by design, the present study focused on CA-HIV living in Uganda, and owing to considerable cultural variation in East Africa, our results may not be generalizable to other countries in the region.

## Conclusions

In summary, approximately 18% of CA-HIV living in Uganda met DSM-5 symptom count plus impairment criteria for neurological disorder. Risk factors of neurological disorders among CA-HIV as hypothesised in the conceptual framework fell under two broad domains of HIV related factors (less viral load suppression at baseline) and socio-demographic factors (age, gender, ethnicity and residence in the rural area). However, the association between neurological disorders on one hand and ethnicity and externalising disorders on the other seem to suggest a genetic vulnerability component. The association between ‘having a neurological disorder’ and earlier age of sexual intercourse, may have important implications for clinical management, quality of life, long-term outcome and possible disease transmission. Moving forward, there is a definite need to integrate neurological/mental health services into routine HIV care for youth in low-and middle-income settings such as those in sub-Saharan Africa. This should include the development of cost effective assessment and treatment strategies that have high probability of success in challenging interventional settings.

## Additional files

Additional file 1: Data collection tools for the study. (DOCX 39 kb)

Additional file 2: Characteristics of study participants. (DOCX 15 kb)

Additional file 3: Correlates of neurological disorders. (DOCX 41 kb)

## Abbreviations

ADD: Attention deficit disorder
ADHD: Attention - deficit / hyperactivity disorder
AIDS: Acquired immune deficiency syndrome
APA: American psychiatric association
ART: Anti-retroviral therapy
CA-HIV: Children and adolescents infected with HIV/AIDS
CASI-4R: Child adolescent symptom inventory-4Revised
CBCL: Child behavioural checklist
CD: Conduct disorder
CDC: Centre for disease control and prevention
CHAKA: stands for the study ‘Mental health among HIV infected CHildren and Adolescents in KAmpala and Masaka, Uganda’
CTX: Cotrimoxazole
DISC-IV: Diagnostic interview schedule for children version IV
GAD: Generalised anxiety disorder
HIV: Human immunodeficiency virus
MDD: Major depressive disorder
ODD: Oppositional defiant disorder
PD: Psychiatric disorder
YI-4R: Youth Inventory-4Revised

## References

[1] Benton TD, Lachman A, Seedat S (2013). HIV/aids. Addressing the mental health needs of affected children and families.

[2] Boivin MJ, Ruisenor-Escudero H, Familiar-Lopez I (2016). CNS impact of perinatal HIV infection and early treatment: the need for behavioral rehabilitative interventions along with medical treatment and care. Curr HIV/AIDS Rep.

[3] Owen JP, Baig B, Abbo C, Baheretibeb Y. Child and adolescent mental health in sub-Saharan Africa: a perspective from clinicians and researchers. Bjpsych International 2016;13 (2). 10.1192/s2056474000001136 PMC5619623 29093899

[4] Bearden D, Steenhoff AP, Dlugos DJ, Kolson D, Mehta P, Kessler S (2015). Early antiretroviral therapy is protective against epilepsy in children with human immunodeficiency virus infection in Botswana. J acquired immune deficiency syndromes (1999).

[5] Benton TD (2010). Psychiatric considerations in children and adolescents with HIV/AIDS. Child Adolesc Psychiatr Clin N Am.

[6] Mpango RS, Kinyanda E, Rukundo GZ, Levin J, Gadow KD, Patel V (2017). Prevalence and correlates for ADHD and relation with social and academic functioning among children and adolescents with HIV/AIDS in Uganda. BMC psychiatry.

[7] Mpango RS, Kinyanda E, Rukundo GZ, Gadow KD, Patel V (2017). Cross-cultural adaptation of the Child and adolescent symptom Inventory-5 (CASI-5) for use in central and South-Western Uganda: the CHAKA project. Trop Dr.

[8] Gadow KD, Chernoff M, Williams PL, Brouwers P, Morse E, Heston J (2010). Co-occuring psychiatric symptoms in children perinatally infected with HIV and peer comparison sample. Journal of developmental and behavioral pediatrics : JDBP.

[9] Nachman S, Chernoff M, Williams P, Hodge J, Heston J, Gadow KD (2012). Human immunodeficiency virus disease severity, psychiatric symptoms, and functional outcomes in perinatally infected youth. Archives of pediatrics & adolescent medicine.

[10] Gadow KD, Sprafkin J. Child & Adolescent Symptom Inventory-5 (Ages 5 to 18 Years) Stony Brook, NY: Checkmate Plus.; 2013 a,b. https://www.checkmateplus.com/product/casi5.htm].

[11] Gadow KD, Kaat AJ, Lecavalier L (2013). Relation of symptom-induced impairment with other illness parameters in clinic-referred youth. J child psychology and psychiatry, and allied disciplines.

[12] Kellinghaus C, Engbring C, Kovac S, Moddel G, Boesebeck F, Fischera M (2008). Frequency of seizures and epilepsy in neurological HIV-infected patients. Seizure.

[13] Pascual-Sedano B, Iranzo A, Marti-Fabregas J, Domingo P, Escartin A, Fuster M (1999). Prospective study of new-onset seizures in patients with human immunodeficiency virus infection: etiologic and clinical aspects. Arch Neurol.

[14] Samia P, Petersen R, Walker KG, Eley B, Wilmshurst JM (2013). Prevalence of seizures in children infected with human immunodeficiency virus. J Child Neurol.

[15] Bower WF, Moore KH, Shepherd RB, Adams RD (1996). The epidemiology of childhood enuresis in Australia. Br J Urol.

[16] Unalacak M, Sögüt A, Aktunc E, Demircan N, Altin R. Enuresis noctuma: Prevalence and risk factors among school age children in northwest Turkey 2004.

[17] Sureshkumar P, Jones M, Caldwell PH, Craig JC (2009). Risk factors for nocturnal enuresis in school-age children. J Urol.

[18] Metzger H, Wanderer S, V. VR. Tic disorders. 2012. In: IACAPAP ( International Association for Child and Adolescent Psychiatry and Allied Professions ) e-Textbook of Child and Adolescent Mental Health 2012 [Internet]. Geneva.

[19] Berkovic SF (2015). Genetics of epilepsy in clinical practice. Epilepsy currents.

[20] Ba-Diop A, Marin B, Druet-Cabanac M, Ngoungou EB, Newton CR, Preux PM (2014). Epidemiology, causes, and treatment of epilepsy in sub-Saharan Africa. Lancet Neurol.

[21] von Gontard A. Enuresis. In: IACAPAP (International Association for Child and Adolescent Psychiatry and allied professions ) e-textbook of Child and adolescent mental health [internet]. Geneva; 2012.

[22] von Gontard A. Encopresis. In: IACAPAP (International Association for Child and Adolescent Psychiatry and allied professions ) e-textbook of Child and adolescent mental health [internet]. Geneva; 2012.

[23] von Gontard A, Schaumburg H, Hollmann E, Eiberg H, Rittig S (2001). The genetics of enuresis: a review. J Urol.

[24] Joinson C, Heron J, Butler U, von Gontard A (2006). Psychological differences between children with and without soiling problems. Pediatrics.

[25] Scahill L, Specht M, Page C (2014). The prevalence of tic disorders and clinical characteristics in children. J obsessive-compulsive and related disorders.

[26] Singer HS (2005). Tourette’s syndrome: from behaviour to biology. Lancet Neurol.

[27] Bland JM, Altman DG. Multiple significance tests: the Bonferroni method. Bmj. 1995;310(6973):170. 10.1136/bmj.310.6973.170 PMC2548561 7833759

