# Supplementary material for: Prevalence, correlates for early neurological disorders and association with functioning among children and adolescents with HIV/AIDS in Uganda
Source: BMC Psychiatry. 2019 Jan 21;19:34. doi: 10.1186/s12888-019-2023-9 (PMC6341558; doi:10.1186/s12888-019-2023-9)
Supplement: Supplementary file 1 — Data collection tools for the study. (DOCX 39 kb) [file 12888_2019_2023_MOESM1_ESM.docx]

**Additional file 1: Description of Data Collection Measures**

|  | **Instrument used** | | **Description** | **Questions or categories (examples)** | **Remarks** | **Reference** |
| --- | --- | --- | --- | --- | --- | --- |
| **Socio-demographic factors** | | | |  |  |  |
| **Child/adolescent** | Structured socio-demographic questionnaire;  Study site, age, gender, ethnicity, highest educational level attained and socio-economic status (SEI) | | Socio-  economic index (SEI)  was  constructed from  commonly  available  household items  in a typical  Ugandan  households, has  previously been  used by this study group | To construct the SEI questions such as the following were used: *does your*  *household have*  *electricity?*  Response:  Yes/no | Administered to caregiver  Has previously  been used by this  study group. | (25) |
| **HIV related factors** | | |  |  |  |  |
| WHO clinical  stage for  HIV/AIDS | WHO Clinical  Staging criteria | |  | Respondents  classified as Stage I  to IV based on the  presence/absence a  combination of 17  HIV associated  clinical symptoms | This was administered to adolescents | (33) |
| CD4 Nidar | Worst CD4 count ever attained | |  | Cells/ µl of blood |  |  |
| CD4 counts | CD4 count undertaken in the last 6 months | |  | Cells/ µl of blood |  |  |
| HIV RNA Viral load | Viral load determined at assessment | |  | Number of copies of HIV RNA/ml of blood |  |  |
| Caregiver HIV status | Inquire of caregiver HIV status | |  | Assessed by asking caregiver about their HIV status? Possible responses 1=Positive 2=Negative 3=Not know |  |  |
| Child/adolescent on ART | Inquire whether CA-HIV is on ART | |  | Assessed by the question, Is this child on ART? Possible responses: *1=Yes, 2=No* |  |  |
| **Non-HIV related factors** | | |  |  |  |  |
| Enough food | One item question | | Closed question | The item was; *In the last month,*  *did you or your*  *family have enough*  *food?*  Response:  Yes/no | This was administered to caregivers  It was Previously  used in the  HIV  situation of  Uganda by  Kinyanda et al., 2011. | (25) |
| Premature birth | One item question | | Closed question | *Was this child born a pre-mature at birth?*  Possible responses:  1= was a premature at birth; 2= was not a premature at birth; 3= I do not know | Caregiver response |  |
| **Adverse Outcomes** | | |  |  |  |  |
| Poor social functioning | | Questions inquired about social functioning |  | *Whether the CA-HIV had suffered disciplinary measures such as suspensions, dismissal in the last term/semester ?*  Response 1=yes, 2=No | Caregiver response | (35) |
| Missed school in last term | | Assessed by means of a question |  | *Number of days missed at school in the last term*  Response  Number…… | Caregiver response | (35) |
| Visits to the health unit in the last month | | Assessed by question |  | *How many times did you visit the health unit in the last month?*  Response  Number ……… | Caregiver response | (35) |
| Admissions to hospital | | Assessed by question |  | *For how many days were you admitted to hospitals in the last 6 month?*  Response  Number …………. | Caregiver response | (35) |
| Onset of early sexual intercourse | | Assessed by question |  | *Have you ever had sex?*  Response  1=Yes 2=No | Adolescent only |  |
| Adherence to ART | | Assessed by question |  | For those on ART: *How many days in the past 3 days have you missed taking ART?*  Response  Number of days missed ………. | CA-HIV | (35) |
