# Supplementary material for: Prevalence, correlates for early neurological disorders and association with functioning among children and adolescents with HIV/AIDS in Uganda
Source: BMC Psychiatry. 2019 Jan 21;19:34. doi: 10.1186/s12888-019-2023-9 (PMC6341558; doi:10.1186/s12888-019-2023-9)
Supplement: Supplementary file 2 — Characteristics of study participants. (DOCX 15 kb) [file 12888_2019_2023_MOESM2_ESM.docx]

| **Variable** | **Level** | **Total**  **(n=1,070)**  **n (%)** | **Children**  **(n=677)**  **n (%)** | **Adolescents (n=393)**  **n (%)** |
| --- | --- | --- | --- | --- |
| **Study Site** | Urban  Rural | 493 (46.1%)  577 (53.9%) | 300 (44.3%)  377 (55.7%) | 193 (49.1%)  200 (50.9%) |
| **Sex** | Male  Female | 520 (48.6%)  550 (51.4%) | 335 (49.5%)  342 (50.5%) | 185 (47.1%)  208 (52.9%) |
| **Religion** | Christian  Muslim  Others/missing | 850 (79.4%)  215 (20.1%)  5 (0.5%) | 536 (79.2%)  138 (20.4%)  3 (0.4%) | 314 (79.9%)  77 (19.6%)  2 (0.5%) |
| **Tribe** | Baganda  Non-Baganda | 775 (72.4%)  295 (27.6%) | 493 (72.8%)  184 (27.2%) | 282 (71.8%)  111 (28.2%) |
| **Child lives with** | Both parent  Single parent  Grandparents  Others/missing | 278 (26.0%)  403 (37.6%)  216 (20.2%)  173 (16.2%) | 198 (29.3%)  261 (38.6%)  144 (21.3%)  74 (10.9%) | 80 (20.4%)  142 (36.1%)  72 (18.3%)  99 (25.2%) |
| **Orphanhood** | Single parent orphan  Double parent orphan  Non-orphan | 386 (36.1%)  121 (11.3%)  563 (52.6%) | 265 (30.8%)  57 (6.6%)  538 (62.6%) | 201 (42.0%)  95 (19.8%)  183 (38.2%) |
| **Highest level of education attained** | No formal  Pre-primary  Primary  Secondary  Missing | 28 (2.6%)  182 (17.0%)  755 (70.6%)  103 (9.6%)  2 (0.2%) | 11 (1.6%)  179 (26.5%)  485 (71.9%)  0 (0%)  2 (0.3%) | 17 (3.6%)  3 (0.6%)  270 (68.7%)  103 (26.2%)  0 (0%) |
| **Socio-economic index** | Mean(Std) | 4.39 (1.8) | 4.20 (1.7) | 4.72 (1.8) |
| **Socio-economic index (grouped)** | 0 – 2  3 – 4  5 – 6  7 – 9 | 150 (14.0%)  392 (36.6%)  397 (37.1%)  131 (12.2%) | 110 (16.3%)  267 (39.4%)  233 (34.4%)  67 (9.9%) | 40 (10.2%)  125 (31.8%)  164 (41.7%)  64 (16.3%) |
| **Baseline CD4 counts (cells / µl)** | <200  200-349  350-599  600-899  ≥900  missing | 49 (4.6%)  58 (5.4%)  187 (17.5%)  262 (24.5%)  501 (46.8%)  13 (1.2%) | 28 (4.1%)  17 (2.5%)  68 (10.0%)  130 (19.2%)  427 (63.1%)  7 (1.0%) | 21 (5.3%)  41 (10.4%)  119 (30.3%)  132 (33.6%)  74 (18.8%)  6 (1.5%) |
| **Child on ART at baseline?** | Yes  No | 1024 (95.7%)  46 (4.3%) | 651 (96.2%)  26 (3.8%) | 373 (94.9%)  20 (5.1%) |

**Additional file 2: Characteristics of study participants**
