# Supplementary material for: Prevalence, correlates for early neurological disorders and association with functioning among children and adolescents with HIV/AIDS in Uganda
Source: BMC Psychiatry. 2019 Jan 21;19:34. doi: 10.1186/s12888-019-2023-9 (PMC6341558; doi:10.1186/s12888-019-2023-9)
Supplement: Supplementary file 3 — Correlates of neurological disorders. (DOCX 41 kb) [file 12888_2019_2023_MOESM3_ESM.docx]

**Additional file 3: Correlates of neurological disorders**

| **Correlates** | **N=1070** | **Enuresis/ Encopresis^†^** | | **Motor/ Vocal Tics^†^** | | **Epilepsy**^‡^ | |
| --- | --- | --- | --- | --- | --- | --- | --- |
|  |  | **Unadjusted OR**  **95% CI,**  **p-value** | **Adjusted OR**  **95% CI,**  **p-value** | **Unadjusted OR**  **95% CI,**  **p-value** | **Adjusted OR**  **95% CI,**  **p-value** | **Unadjusted OR**  **95% CI,**  **p-value** | **Adjusted OR**  **95% CI,**  **p-value** |
| **Child socio-demographic factors** | | |  |  |  |  |  |
| ***Site***  Urban  Rural | 493  577 (53.9) | 1  2.38 (1.53, 3.70)  ***P < 0.001*** | 1  2.25 (1.42, 3.54)  ***P < 0.001*** | 1  0.82 (0.48, 1.39)  ***P = 0.5*** |  | 1  0.98 (0.53, 1.81)  ***P = 0.9*** |  |
| ***Age*** (per year) |  | 0.78 (.73, .85)  ***P < 0.001*** | 0.79 (0.73, 0.85)  ***P < 0.001*** | 1.28 (1.18, 1.40)  ***P < 0.001*** | 1.28 (1.17, 1.40)  ***P < 0.001*** | 1.02 (0.93, 1.11)  ***P = 0.7*** |  |
| ***Gender***  Female vs Male | 550 (51.4) | 0.75 (0.50, 1.11)  ***P = 0.16*** |  | 1.80 (1.03,3.15)  ***P = 0.04*** |  | 1.47 (0.79, 2.74)  ***P = 0.2*** |  |
| ***Ethnicity***  Baganda  Non-Baganda | 775  295 (27.6) | 1  0.58 (0.35, 0.96)  ***P = 0.03*** | 1  0.56 (0.33, 0.95)  ***P = 0.03*** | 1  0.35 (0.16, 0.79)  ***P = 0.01*** | 1  0.33 (0.14, 0.76)  ***P = 0.01*** | 1  1.76 (0.94, 3.30)  ***P = 0.08*** | 1  1.87 (0.98, 3.57)  ***P = 0.06*** |
| ***Social economic status***  Median score (IQR) | 4  (3-6) | 0.88 (0.78, 0.98)  ***P = 0.02*** |  | 1.07 (0.92, 1.25)  ***P = 0.4*** |  | 0.96 (0.80, 1.13)  ***P = 0.6*** |  |
| ***CA-HIV highest Educational level attained***  Secondary or more versus Primary or none | 412 (39.2) | 0.73 (0.48, 1.12)  ***P = 0.15*** |  | 1.12 (0.65, 1.95)  ***P = 0.7*** |  | 0.80 (0.41, 1.54)  ***P = 0.5*** |  |
| **HIV- related factors** | |  |  |  |  |  |  |
| ***CD4 nadir***  <200  200-349  350-599  600-899  >=900 | 138 (13.9)  126 (12.7)  204 (20.5)  254 (25.5)  273 (27.4) | 1  1.40 (0.53, 3.67)  1.57 (0.66, 3.73)  2.34 (1.05, 5.24)  2.39 (1.08, 5.30)  ***P = 0.10*** |  | 1  0.40 (0.14, 1.15)  0.82 (0.38, 1.76)  0.39 (0.17, 0.92)  0.33 (0.14,0.78)  ***P = 0.04*** |  | 1  0.42 (0.13, 1.37)  0.32 (0.11, 0.96)  0.63 (0.27, 1.51)  0.49 (0.20, 1.20)  ***P = 0.3*** |  |
| ***Current CD4***  Median  (IQR)  CD4 cell count per 100 cells | 856*  (578,1203) | 1.05 (1.01, 1.08)  ***P = 0.01*** |  | 0.93 (0.87, 0.99)  ***P = 0.02*** |  | - 1. (0.97, 1.06)   ***P = 0.6*** |  |
| ***Baseline viral load*** (copies/ml): median (IQR)  VL < 200  (suppressed versus not suppressed) | 20 (116 - 18268)  66.8% | 1.71 (1.07, 2.73)  ***P = 0.02*** | 1.75 (1.08, 2.83)  ***P = 0.02*** | 1. (0.91, 1.10)   ***P = 0.9*** |  | 1.03 (0.54, 1.97)  ***P = 0.9*** |  |
| ***Child on ART***  Yes vs No | 1024 (95.7) | 0.62 (0.19, 2.02)  ***P = 0.4*** |  | 0.38 (0.05, 2.84)  ***P = 0.4*** |  | 1.09 (0.26, 4.65)  ***P = 0.9*** |  |
| ***CA-HIV worst WHO stage***  Stage 1  Stage 2  Stage 3 | 128 (12.0)  596 (55.7)  346 (32.3) | 1  1.24 (0.65, 2.37)  0.82 (0.40, 1.67)  ***P=0.2*** |  | 1  0.50 (0.24, 1.05)  0.62 (0.29, 1.34)  ***P = 0.2*** |  | 1  1.03 (0.39, 2.76)  1.04 (0.37, 2.94)  ***P = 0.9*** |  |
| ***Caregiver HIV status***  Negative  Positive  Don’t know/missing | 325 (30.4)  692 (64.7)  53 (5.0) | 1  1.12 (0.72, 1.74)  0.57 (0.17, 1.93)  ***P=0.4*** |  | 1  0.97 (0.53, 1.75)  1.89 (0.67, 5.35)  ***P = 0.4*** |  | 1  1.43 (0.69, 2.96)  1.89 (0.50, 7.11)  ***P = 0.5*** |  |
| **Non- HIV related factors** | |  |  |  |  |  |  |
| ***Child born Premature***  Yes vs No | 19 (1.8%) | 0.50 (0.07, 3.75)  ***P = 0.5*** |  | N/a |  | 1.33 (0.17, 10.23)  ***P = 0.8*** |  |
| ***Food insufficiency***  ***In last month***  Yes versus no | 857 (81.2) | 0.83 (0.50, 1.39)  ***P = 0.5*** |  | 1.51 (0.82, 2.75)  ***P = 0.18*** |  | 1  1.63 (0.83, 3.19)  ***P = 0.15*** |  |

Note:

*Analysis using logistic regression at 6months.

†Adjusting for variables that were significant in the group models in final model including age, sex and site.

‡Adjusting; none of the factors were independently significantly associated with epilepsy in the group models.
